# RAPID framework for improved access to precision oncology for lethal disease: Results from a modified multi-round delphi study

**DOI:** 10.3389/frhs.2023.1015621

**Published:** 2023-02-27

**Authors:** Kristin Bright, Anneliese Mills, John-Peter Bradford, David J. Stewart

**Affiliations:** ^1^ Department of Anthropology, Middlebury College, Middlebury, VT, United States; ^2^ Department of Anthropology, University of Toronto, Toronto, ON, Canada; ^3^ Department of Family and Community Medicine, University of Toronto, Toronto, ON, Canada; ^4^ Life-Saving Therapies Network, Ottawa, ON, Canada; ^5^ Department of Medicine, University of Ottawa, Ottawa, ON, Canada

**Keywords:** precision oncology, delphi method, clinical trials, novel therapeutics, lethal cancers, cancer policy and governance, barriers and facilitators, system change

## Abstract

**Introduction:**

Predictive oncology, germline technologies, and adaptive seamless trials are promising advances in the treatment of lethal cancers. Yet, access to these therapies is stymied by costly research, regulatory barriers, and structural inequalities worsened by the COVID-19 pandemic.

**Methods:**

To address the need for a comprehensive strategy for rapid and more equitable access to breakthrough therapies for lethal cancers, we conducted a modified multi-round Delphi study with 70 experts in oncology, clinical trials, legal and regulatory processes, patient advocacy, ethics, drug development, and health policy in Canada, Europe, and the US. Semi-structured ethnographic interviews (*n* = 33) were used to identify issues and solutions that participants subsequently evaluated in a survey (*n* = 47). Survey and interview data were co-analyzed to refine topics for an in-person roundtable where recommendations for system change were deliberated and drafted by 26 participants.

**Results:**

Participants emphasized major issues in patient access to novel therapeutics including burdens of time, cost, and transportation required to complete eligibility requirements or to participate in trials. Only 12% of respondents reported satisfaction with current research systems, with “patient access to trials” and “delays in study approval” the topmost concerns.

**Conclusion:**

Experts agree that an equity-centered precision oncology communication model should be developed to improve access to adaptive seamless trials, eligibility reforms, and just-in-time trial activation. International advocacy groups are a key mobilizer of patient trust and should be involved at every stage of research and therapy approval. Our results also show that governments can promote better and faster access to life-saving therapeutics by engaging researchers and payors in an ecosystem approach that responds to the unique clinical, structural, temporal, and risk-benefit situations that patients with life-threatening cancers confront.

## Introduction

Predictive oncology, gene-targeted therapies, and germline technologies have the potential to wholly transform cancer treatment (1–3). Cross-institutional studies such as I-PREDICT demonstrate how trial paradigms that match specific driver mutations with particular agents are correlated with improved disease control and overall survival and may be optimized to treat molecularly complex cancers (4). Immunotherapies like mRNA vaccines are another promising frontier (5) as well as therapeutic approaches beyond first-line immunotherapies including KRAS and PARP inhibitors, CAR T-cell therapy, and signaling pathways such as the hypoxia-adenosine axis (6, 7). Worldwide, however, access is stymied by inefficient research and regulatory barriers that impede progress and block access (8–12). Clinical researchers describe a bureaucratic burden that has grown exponentially in recent years, impeding not only the sustainability of research but the capacity to get trials open in the first place, and dampening the enthusiasm of junior investigators (13, 14). The COVID-19 emergency has worsened disruptions in cancer treatment across the board, exposing and creating new forms of structural and clinical disorder (15–17). While hospital closures and postponement of care led to a temporary drop in cancer diagnoses overall in 2020–21, studies show that an uptick in more advanced diagnoses and mortality is already evident (18).

Despite global disruptions in care, the fight against COVID could be a catalyst for speeding up access to precision therapies and reducing cancer death (15, 16). In contrast to the lightning speed by which COVID-19 vaccines were tested and rolled out, it currently takes 12 years and US $2.6 billion on average to bring a new drug from discovery to market (19). Only 5% of new agents that enter trials are eventually approved for marketing, although the recent designation of “breakthrough drugs” has permitted faster approval at lower cost (20). Moreover, drug discovery is disproportionate across cancer type. In 2020, registration trials were roughly proportional to lethal cancer incidence but not mortality burden, revealing a mismatch between burden of death and potential new therapies in the pipeline (21). As the prevalence of a treated disease decreases, the median cost and negative recommendation rate for a drug go up, making therapies particularly expensive for rare diseases (22–24). One in twelve North Americans will be diagnosed with a rare disorder. Yet, promising therapies for these diseases fall by the wayside (25, 26). One study of effective new therapies estimated that a median of 523,890 life-years could be saved worldwide, per drug example, if the time to approval was 5 instead of 12 years and if all relevant patients could access therapy (27).

There is currently no comprehensive guidance in place for rapid, equitable access to targeted therapies for lethal cancers. The purpose of this study was to take steps towards the identification of such guidance, through the characterization of major barriers and facilitators in access to precision therapies as perceived by 70 experts in cancer treatment, clinical trials, patient advocacy, research ethics, legal and regulatory processes, health economics, and public policy in Canada, Europe, and the US. The goal was to develop research and regulatory policy guidance for improved, just-in-time access to breakthrough therapies. Despite wide-ranging professional and geographical differences among the experts, the recommendations arrived at by specialists in this study offer internationally germane guidance. The study concludes with a synthesis of those results and recommendations in the form of Rapid Access to Precision Informatics and Drugs or the “RAPID” framework.

## Methods

A modified multi-round Delphi approach was used to investigate the opinions of 70 experts regarding barriers and opportunities in precision oncology research trials and therapeutics. The Delphi method was developed by RAND in the 1950s for futures forecasting and involves statistical calculation of group opinion through multiple rounds of questionnaires that gradually aggregate disparate views into a convergence of opinion (28). Health services researchers have combined the Delphi method with participatory action research (PAR) to involve healthcare leaders in a problem and its resolution as opposed to simply collecting their opinions (29). This study adopted the modified Delphi-PAR approach to carry out three rounds of data gathering, analysis, and consensus building, from January 2018 to January 2020. Ethics approval was obtained from the University of Toronto REB.

### Round 1/interviews

124 individuals were identified *via* purposeful sampling based on minimum 10 years' expertise in high-level oncology research and policy in Canada, Europe, and the United States. All of those sampled are leaders and directors in oncology research, government regulatory agencies, industry, and provincial and national health policy. An interview sample of 40 individuals was purposefully created from the parent list, to insure a range of professional sectors, geographies, work experience, and engagement in national and international cancer policy. A total of 33 experts participated in semi-structured interviews lasting one hour. 4 interviews were conducted in person, 2 *via* Skype, and 27 by telephone. 19 participants were based in Canada, 4 in Europe, and 10 in the US. Participants were distributed across professional sectors including oncology/precision medicine (8 interviewees), research ethics and trials administration (4 interviewees), patient advocacy (6 interviewees), health economics and data science (3 interviewees), pharma industry (4 interviewees), regulatory and approval processes (3 interviewees), and public affairs and health policy (5 interviewees).

**Figure 1:**
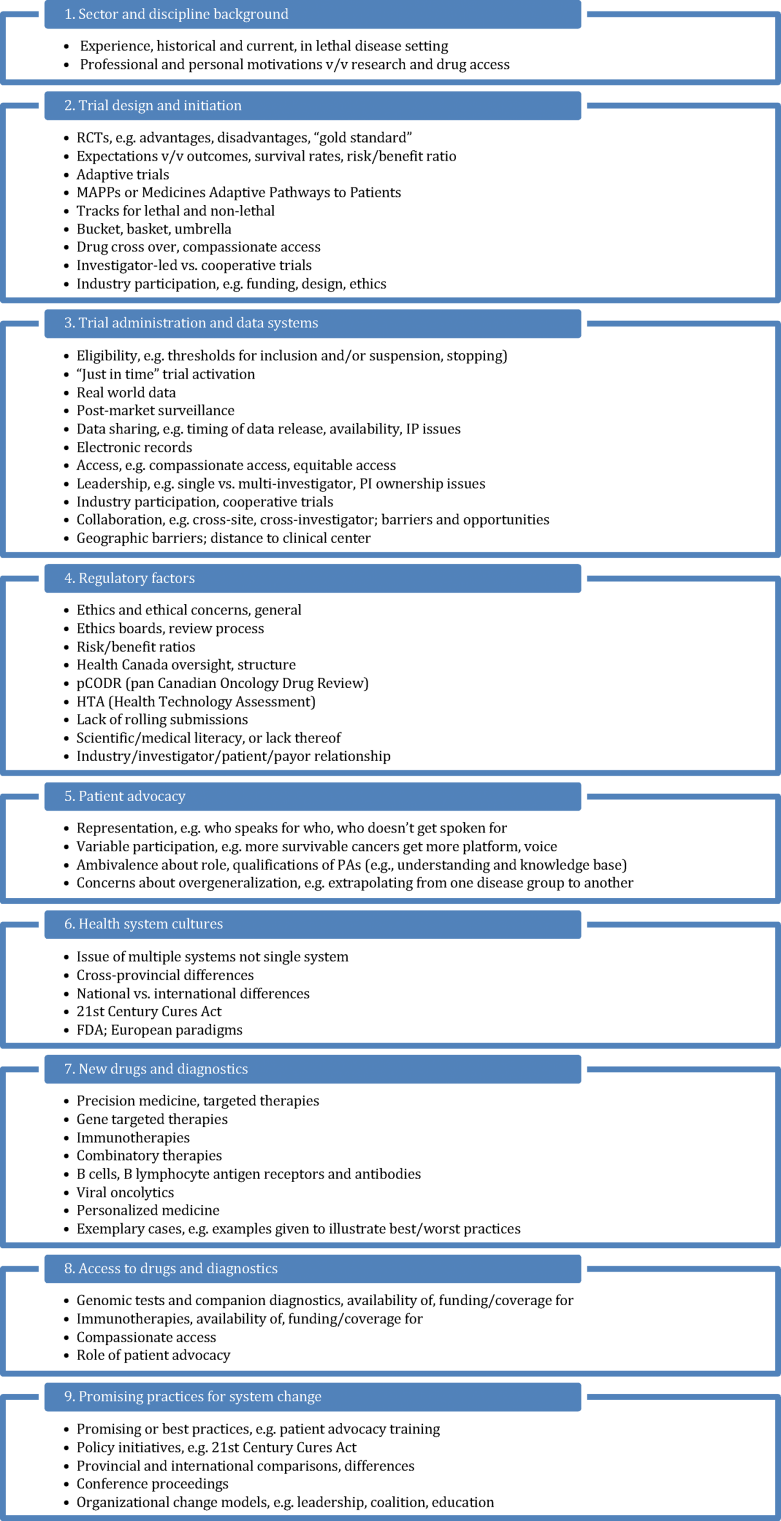
Primary and secondary thematic codes.

Participants provided verbal consent prior to participation, and permission was obtained for audio-recording. Written informed consent was not required in accordance with institutional requirements. Analysis: Qualitative thematic analysis was performed on the first five transcripts for identification of salient issues and repetitions (30). The constant comparison method was used to characterize 9 primary and 57 secondary codes (Figure 1). Codes were then used to thematically analyze all 33 transcripts (31, 32). Due to the diversity of scientific and occupational backgrounds represented in the sample, thematic saturation occurred at 30 interviews. Saturation is the point at which little or no new information, ideas, or opinions appear (33) and the most salient or culturally important information has been obtained and categorized (34). Codes were then utilized to inform the survey questions and menu options (Round 2) and a shortlist of topics for the policy roundtable (Round 3).

### Round 2/survey

Barriers and facilitators identified during Round 1 (Figure 1) were used to design a simple survey with three sections: barriers to research, barriers to therapy approval, and facilitators for improved access. The survey was built using the SurveyMonkey cloud-based platform and pilot tested with four volunteers for clarity, format, and time duration. After some minor edits for wording and flow, the survey was finalized and the full parent sample (*n* = 124) was invited including the 33 participants from Round 1 to participate in a 10-minute survey on barriers and access to therapeutics for lethal disease. All invitees were sent one invitation with a link to the survey and two reminders *via* email. No incentive or gift was offered for participation. 47 individuals completed the survey for a response of 38%. Participants were first asked demographic questions regarding sector of expertise and country of residence. Respondents were asked to then select “all that apply” from one pull-down menu “barriers in research” and a second pull-down menu “barriers in drug approvals.” Items on each menu had been obtained during Round 1 analysis. Text boxes invited participants to elaborate on issues not listed. Respondents also completed ranking questions after each “barriers” question, where they prioritized the “top three issues.” Lastly, participants were asked to select from a pull-down menu “steps for change,” also with options identified during Round 1. Again, fillable boxes were offered for the elaboration of strategies not mentioned in the pull-down. Throughout, respondents were given the option to select “N/A; not able to comment” and “I see no problem with the current system.” *Quantitative analysis*: SurveyMonkey software was used to generate basic frequencies, averages, and categorized views of text responses (see Results). *Qualitative analysis*: we co-analyzed Round 1 interview and Round 2 survey data using the same qualitative comparison and thematic analyses used in Round 1, to create a shortlist of topics for Round 3 (Roundtable).

### Round 3/roundtable

In the final round of study, a total of 26 individuals were invited from the original parent sample (*n* = 124). 21 of these experts had previously participated in the interview or survey groups. The participation of 5 new experts brought the overall study participant total to 70 unique individuals. The process for invitation was purposeful, with the primary goal being a diverse group based on work sector, experience in lethal cancer therapeutics, and geography. The fact that the roundtable was hosted in-person in Ottawa created some limitation with regard to geography, and several invitees originally invited from other countries declined. In the end, 16 participants were from Canada and 10 from the US.

The roundtable was a 2-day event in Ottawa, Ontario, and was hosted by the non-profit cancer patient advocacy organization Life-Saving Therapies Network (LSTN). Audio and written observations were collected during the plenary sessions “Challenges and Issues in Research and Research Protocols,” “Challenges and Issues in Regulatory Approval Policy and Processes,” and “Synthesizing Research and Regulatory Recommendations” as well as breakout sessions. Qualitative analysis: notes and transcriptions were compiled and analyzed through a 4-part coding schema based on four domains: the problem headlining the session; specific barriers identified under that problem; opportunities for change including unused and under-used practices; and recommended actions for system change. Next, coded Round 3 data were examined alongside data from Rounds 1 and 2 for any new or differently emphasized information. Lastly, we assembled our findings in the Rapid Access to Precision Informatics and Drugs (RAPID) framework, paying particular attention to participants' reactions during the third roundtable plenary “Synthesizing Research and Regulatory Recommendations” including recommendations increasingly emphasized across all rounds of study.

## Results

### Interview results

Following transcription and constant comparison analysis, 9 primary areas and 57 sub-areas of characterization emerged (Figure 1). From those coded findings, we further processed the interview data to arrive at **five system performance issues**: access and equity, research trial re/design, leveraging data science and informatics, patient advocacy, and regulatory reform.

#### Access and equity

Patient access to novel phase I/II clinical trials was the top concern. Participants further characterized the need for: rapid identification of patients eligible for trials targeting rare tumor subgroups; better digital support for patients to navigate unfriendly platforms for available trials; rapid referral of relevant candidates to clinical investigators; reduction of physical barriers (most trials require patients to live close to a treatment center, excluding patients who live outside large urban centers); and reduction of time, resources, and transportation required of patients and caregivers simply to complete eligibility screening. Interviewees highlighted inequalities in compassionate access programs for agents that have demonstrated efficacy but are not yet fully approved. Furthermore, an effective new drug may be approved and funded in one country or state/province but not in another.

#### Trial re/design

Participants resoundingly agreed it takes too long to activate trials. Preclinical toxicology evaluation, one respondent explained, adds little value, and involves “excessive steps” from initial clinical trial concept to trial activation. To address these barriers, participants highlighted the need for “just-in-time” trial activation (see below) where physicians and their patients can rapidly access trials already approved elsewhere, even when that trial is not yet approved locally.

Most participants agreed that emphasis on randomized controlled trials (RCTs) as the gold standard is a major barrier to expedited access to new therapies. Recruitment of sufficient patients to statistically power RCTs is a major hurdle for uncommon diseases and tumor types. RCTs do not adequately consider that the most common unrecognized subpopulations may determine which therapy is judged to be most effective, while another therapy may be superior in smaller unrecognized subpopulations. As an alternative to three-stage RCTs, the recent move to expedite approval of highly effective drugs through “breakthrough drug” designation is a major step forward. Participants also noted that new trial designs such as “umbrella,” “basket,” “adaptive,” “seamless,” and others (35–39) are a positive development.

Participants explained that study eligibility criteria are often inappropriately narrow. This limits patient access to trials while markedly slowing accrual and making it uncertain whether trial results may be generalized to the population. Participants noted the importance of patients being permitted to crossover to the alternate trial arm if their designated therapy was not working for them. They also stressed the need for real-world data and predictive models in trial science, an area of concern that overlaps with the next priority issue.

#### Leveraging data science and informatics

Redundant or high-volume adverse event (AE) reporting was described as a major barrier to scientific research. Clinical researchers repeatedly described AE reports (typically sent *via* email and requiring a sign-off) as the most unpleasant part of their day. The generation and tracking of these data have become increasingly onerous and expensive. Inappropriately excessive AE reporting also increases the chance of an investigator missing an important alert that is buried in an avalanche of unimportant alerts.

Experts also pointed to data deserts and data silos, or instances where there are insufficient data or where proprietary interests create barriers. Although most patients are open to having their data shared for appropriate research purposes, inappropriately restrictive privacy policies are a major barrier. There are also painfully under-used opportunities for employing real-world data once a new therapy is approved. For example, in Canada, huge amounts of health care data are collected, but these are generally not used by Health Canada for regulatory purposes or by provinces for reimbursement decisions.

One participant suggested a repurposing of current systems through algorithmic science. “Earlier regulatory approval […] could be accomplished if CADTH [Canadian Agency for Drugs and Technologies in Health] were willing to make computational analysis part of the approval process. And the institution of DSEN [Drug Safety and Effectiveness Network] or something analogous to it, is quite possibly the correct institution to help.”

#### Patients at the table

Most participants agreed that a lack of public awareness regarding precision therapies has stymied progress and patient advocates are well-positioned to solve this issue. There was strong support for a more unified and globalized patient advocacy movement to expedite access to therapies for malignant diseases. Several individuals focused on training, i.e., giving advocates skills for targeted participation in research design, methodology, and translation of data to the public. As one respondent stated, “Advocates should be treated as people with variable skillsets [and should not be recruited] simply to fill a quota.” Additionally, some stakeholders believe that “adding complexity will always improve safety,” but do not appreciate that complexity can markedly slow progress while adding little value.

Another participant drew a distinction between patient advocates for lethal and non-lethal malignancies. They explained that some advocacy groups for non-lethal cancers were opposed to the US 21st Century Cures Act, viewing it as a way for companies to earn more money or avoid safety concerns by fast-tracking therapies. Others explained that patients with earlier stage or more treatable diseases tend to focus their advocacy on trials that target incremental reductions of morbidities rather than overall survival. In contrast, patients with lethal cancers and advocates generally regard therapeutic efficacy, survival, and even the small possibility that a therapy might work as more important than toxicity or risk.

#### Regulatory redirect

Participants observed regulatory barriers at every level: local, state, national, international. A decade ago, one respondent explained, eighteen of her twenty research staff were dedicated to organizing trials and interacting with patients. Now nearly half are “entirely diverted” to regulatory work. Prior to our interview at 10am, this participant had already received nearly 100 emails regarding consent forms and side effects.

Most interviewees stressed the need for regulatory harmonization at the international level. There are critical gaps between what regulators will accept in the case of phase II data, for example, and what regulators require before making recommendations for funding. In Canada, these processes and timelines differ across provinces. As one participant explained: “There is a three-year delay on average, between when any country internationally approves a cancer drug and when that drug becomes available to 80% of Canadians via provincial reimbursement.” It has been calculated that thousands of Canadian life-years are lost due to this delay in access to effective new therapies (40).

Participants also emphasized that regulatory reform is not commensurate with regulatory laxity. Oversight, one person noted, is in place for historical reasons, to protect against the human rights abuses that took place in Nazi concentration camps and the Tuskegee syphilis trial, and to protect against unscrupulous professionals who exploit patients. Other participants expressed concern that research decisions and progress are more influenced by ethics committees than researchers or patients. As another expert explained, “It's become extremely paternalistic […] They think they’re protecting patients, but the patients are desperate to have access to experimental drugs and to be experimented on. The rules to protect them have gotten in the way of early access to experimental treatments.” They emphasized overgrown bureaucracy and paper trails as a priority issue. “There are 20-page consent forms that patients don’t read because there is too much information.”

Many interviewees observed that improvement in this area will require regulatory reform champions. “It may be that there's just one or two places that have the bravery to take on a different type of clinical trial and prove it works,” explained one, “You not only need brave investigators and patients. You need a brave ethics board to approve it.”

### Survey results

Survey participants (*n* = 47) were first asked to report where they live. 89% selected Canada and 11% the US. When asked to “select all that apply” for “which sector(s) do you work in or have you worked in previously,” respondents indicated research (50%), patient advocacy (50%), clinical care (17.4%), health economics (17.4%), drug industry (17.4%), and government (15.2%). Under “other work,” respondents added health technology assessment, not-for-profit, litigation law, human subject protection, and academia. Next, participants reported on perceived barriers and facilitators in precision oncology research and novel therapeutics access. In line with the qualitative results presented in Round 1, survey results are organized in three categories: access and equity; drug approval timing and access; and strategies for system change.

#### Research system access and equity

When asked about their satisfaction with research systems for lethal diseases, only 11.6% said they were “satisfied.” 43.2% responded “neither satisfied or dissatisfied,” 31.8% reported “dissatisfied,” and 4.6% said “highly dissatisfied.” 9.1% declined to comment.

**Figure 2:**
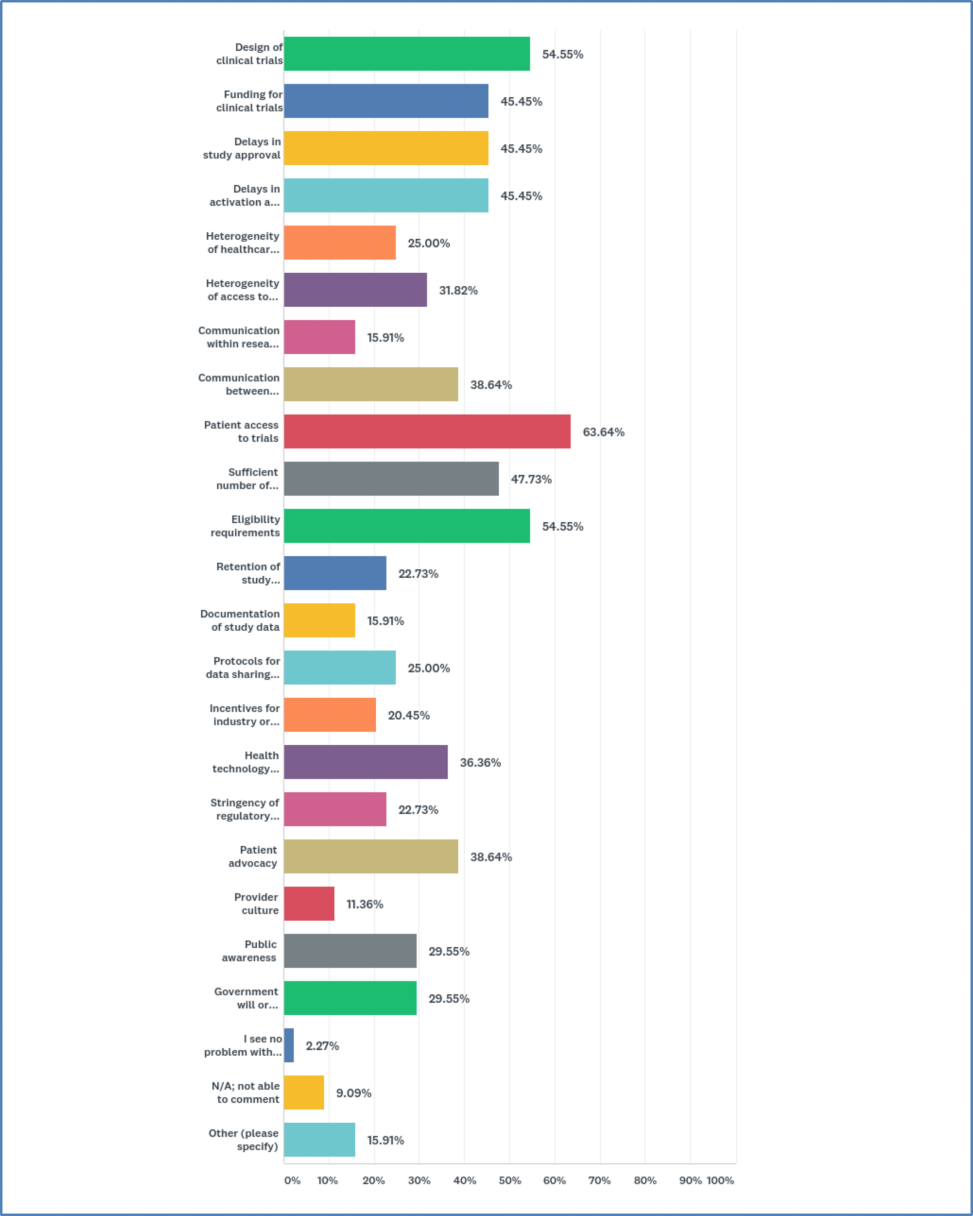
Most problematic areas in research systems for precision oncology.

Participants were asked to select areas they consider problematic. 21 options were offered with the instruction to check all that apply. A majority reported “patient access to trials” (63.6%). Many were also concerned about “design of clinical trials” (54.6%), “eligibility requirements” (54.6%), “sufficient number of patients for trials” (47.7%), “funding for clinical trials” (45.5%), “delays in study approval” (45.5%), and “delays in activation at study site” (45.5%) (Figure 2).

When asked to rank their top three concerns, most reported “patient access to trials” and “delays in study approval” as #1 (two-way tie). The #2 concern was “eligibility requirements,” and #3 was a three-way tie between “design of clinical trials,” “funding for clinical trials,” and “delays in activation at study site.” Respondents offered a range of explanations. Several explained that access is a “heterogeneous” concept that goes beyond whether a clinical rial is “open” to enrollment: access entails availability and legibility of information, and social, financial, and transportation support. Some individuals stressed the need for patient input on study objectives and design.

Other respondents amplified the need for greater government commitment to clinical trials, particularly for rare diseases. There is relatively little government involvement in trials, and most are industry funded. This increases the risk of bias, while leaving a rare disease without a champion if there is no industry interest. Several participants elaborated on the need for “patient engagement in development, implementation, monitoring and evaluation, post approval surveillance, and knowledge translation” and “uncertainty regarding regulation of companion diagnostic or predictive biomarker testing.” One respondent in the US wrote that “state insurance regulators know essentially zero about precision medicine and do not engage with research systems at any level.”

#### Drug approval timing and access

Only 13.5% of respondents said they were “satisfied” with current drug approval systems. 24.3% answered “neither satisfied or dissatisfied,” 35.1% were “dissatisfied,” and 18.9% were “highly dissatisfied.” Combined, 54.1% expressed dissatisfaction.

**Figure 3:**
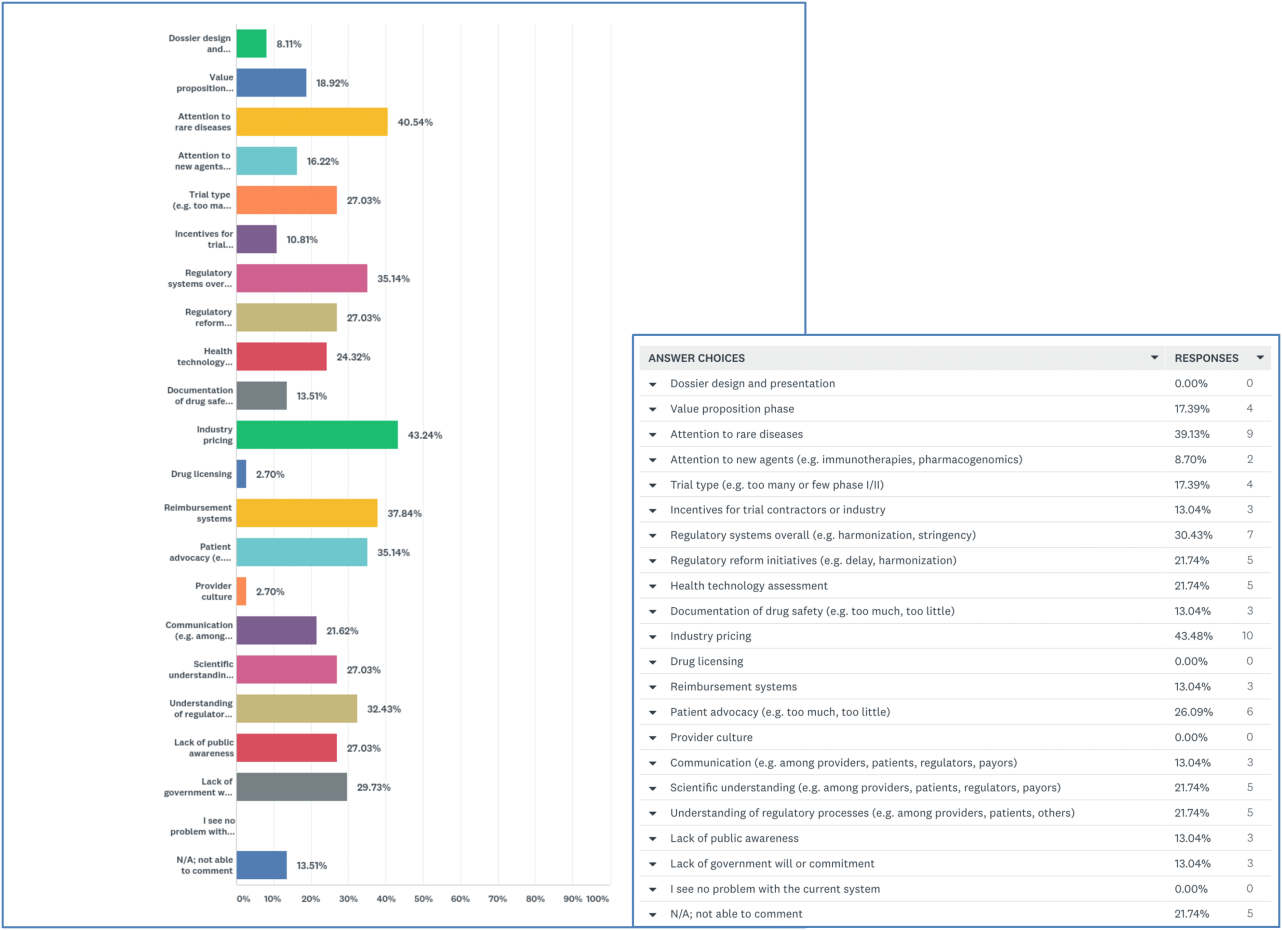
Most problematic areas in approval processes for precision oncology.

When presented with a list of 20 potential issues involved in drug approval and asked to click on problematic factors, 43.2% selected “industry pricing” and 40.5% selected “attention to rare diseases.” Participants expressed concern about “reimbursement systems” (37.8%), “regulatory systems overall” (35.1%), “patient advocacy” (35.1%), and “understanding of regulatory processes” (32.4%) (Figure 3).

Participants ranked “Regulatory systems overall” as their #1 concern followed by “industry pricing” at #2 and “attention to rare diseases” at #3. When asked to elaborate, participants noted the need for regulatory harmonization and flexibility. One person explained that Health Canada might expedite reviews if they accepted reviews completed by the Food and Drug Administration (FDA) or European Medicines Agency (EMA). Other respondents decried Health Canada's abandonment of the Orphan Drug Framework and explained that regulators do not have the expertise to make decisions about rare diseases. Some respondents put the onus on patient advocates and argued that advocates for rare diseases were not effective in presenting a united message to the government.

Lastly, respondents lamented a system where clinical trial organizations (CTOs) are incentivized to create bureaucracy and more business for themselves. As one person explained “There are too many organizations that make a living off ensuring the complexity of the system.” This view echoed observations made in the interviews.

#### Pathways for system change

**Figure 4:**
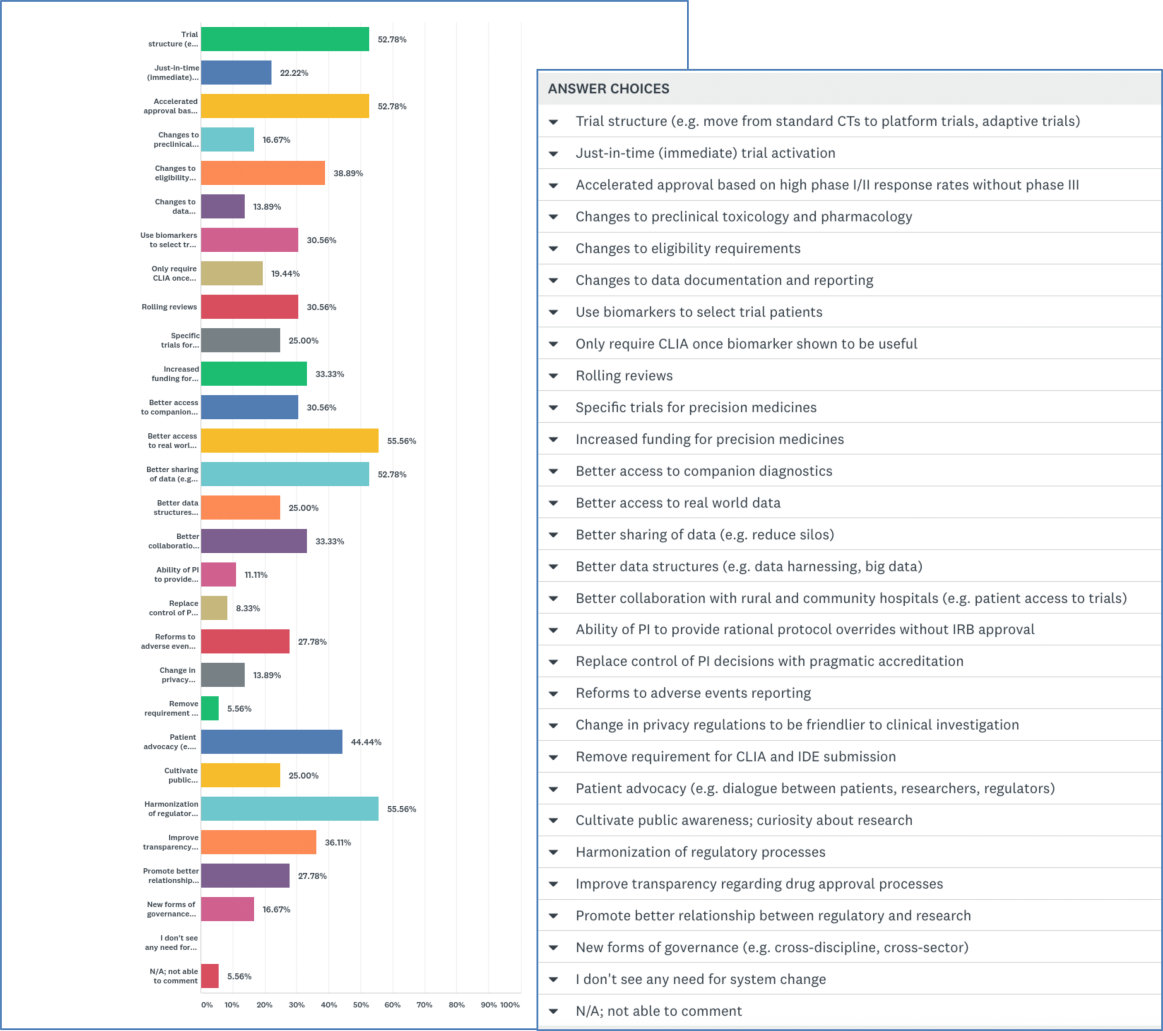
Target areas for system change in precision oncology.

Lastly, respondents were presented with 27 targets for system change with the instruction to “select all that apply.” A majority selected “better access to real world data” (55.6%) and “harmonization of regulatory systems” (55.6%). Many participants also chose “trial re-structuring” (52.8%), “accelerated approval based on high phase I/II response rates without phase III” (52.8%), “better data sharing (reduce silos)” (52.8%), and “patient advocacy (dialogue between patients, researchers, regulators)” (44.4%) (Figure 4).

Respondents were asked to rank the “3 highest priority areas” for system change. Most identified “trial structure (e.g., move from standard clinical trials to adaptive trials)” as their #1 target. Second highest priorities were a tie between “better sharing of data (reduce silos)” and “better access to real world data.” And third-ranked priorities were “accelerated approval based on high phase I/II response rates without phase III” and “improve transparency regarding drug approval processes.”

When invited to elaborate, many respondents noted why trial restructuring should be the first focus. Effective drugs could be approved more rapidly based on a high level of efficacy demonstrated in phase I/II trials conducted in patients identified by relevant predictive biomarkers. Adaptive trials could be particularly efficient, respondents noted; and post market, real-world evidence could confirm the efficacy of therapies approved without a phase III trial. Participants emphasized the need for government regulators and payors to have a better understanding of new trial designs and endpoints for rare diseases (akin to continuing medical education for regulators/payors). Breaking down data silos should be an urgent concern, others noted, with better access to and utilization of post-market data. Others observed that eligibility criteria are often based more on historical reasons than valid safety reasons, with major consequences for the generalizability of trial results.

Finally, participants stressed the need for patient centered communication and transparency at every stage of the approval process, particularly with respect to which trials are open to patients, how patients can best participate, and supportive resources including patient navigation, bus/train vouchers, and a slowed-down, patient friendly approach to hospital orientation, maps and signage in translation, and real-time translation for non-English speakers.

### Roundtable results

**Figure 5:**
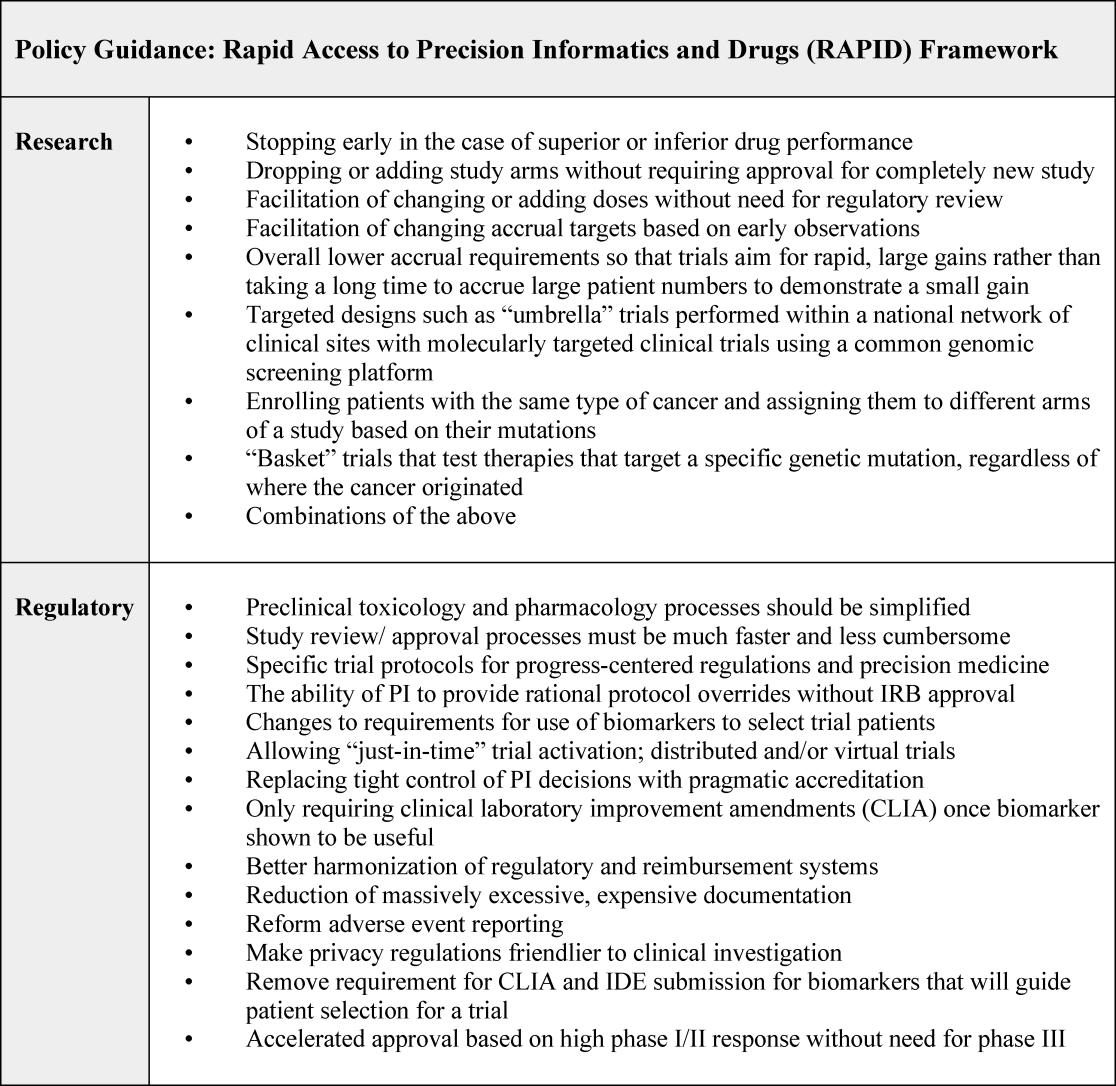
Rapid access to precision informatics and drugs (RAPID).

Roundtable results echoed Round 1 and Round 2 findings while providing further characterization of policy guidance. Figure 5 summarizes our results as the Rapid Access to Precision Informatics and Drugs or “RAPID” framework. Some of these steps are already being effectively used in specialty centers and need to be scaled nationally: for example, adaptive seamless trials; expansion of trial accrual based on initial observations; and approval of agents *via* “breakthrough drug” designation based on high response rates in phase I/II trials (35–37). While some findings reported in the roundtable are variable and contingent on regional and national healthcare settings, most have international relevance since common issues can slow progress across jurisdictions.

## Discussion and conclusion

The findings in this study resoundingly point to the need for rapid and equitable access to precision oncology drugs and predictive technologies. They underscore the need for global guidance regarding how novel agents and technologies are made available. In the view of the experts who took part in this research, a global ecosystem for lethal disease therapeutics should be capacious and multidisciplinary, leveraging big data and AI [data sharing platforms such as GENIE (41), computational modelling, and predictive targets], internationalization of patient advocacy, scientific literacy for patient advocates (empowering them to take part in scientifically complex studies), and the acceleration of approvals of new agents cross-jurisdiction. Roundtable participants excitedly noted several strategies to build upon: adaptive trials that allow researchers to analyze accumulating data at various points in the study; uses of the same data to modify treatment for an individual or the trial itself; reforms to eligibility and stopping criteria to make trials accessible to more patients; and just-in-time trial activation through which a physician seeing a new patient with an uncommon disease could quickly activate a trial already approved in another jurisdiction without getting bogged down in local activation that typically takes a minimum of three to six months.

Overall, participants underlined the need for equity-focused, patient-centered communication that centers transparency and partnership among clinicians, patients, advocates, regulators, and payors. Access is not simply a question of being at the research or policy table; it is the way stakeholders feel involved at each stage. For example, building trust in drug efficacy and safety and assuring patients of data privacy and responsible use of health records, lab data, prognostic data, and post-market data. Experts agreed that more effective, coordinated patient participation in the realm of rare diseases is needed to overcome a government “divide and conquer” approach. “Conversations with patients about research must be our mandate,” one clinician explained. “Regardless of all the regulatory issues, if we had patients clamouring for [trials], we would get them done quicker.” This sentiment is consistent with international research in targeted therapies for lethal disease (42, 43).

The 21^st^ Century Cures Act should be a global starting point because the law is designed to speed the introduction of new treatments by leveraging real-world evidence including observational studies, insurance claims data, and patient feedback. At the same time, informed, evidence-based patient advocacy organizations are a critical mobilizer of patient trust, inclusivity, and equity and should be consulted along the full continuum of research and drug approval. Experts in this study agreed advocacy organizations must be better informed, included, and networked into global research.

Lastly, governments should promote collaboration among advocates, researchers, regulators, and payors through an ecosystem model that responds to the specific biological-clinical, informational, and risk-benefit needs and conditions that patients with life-threatening cancers confront. Above all, medical communities need to prioritize pragmatism. As one participant emphasized in an interview: “all these problems are solvable. We need to reach the tipping point that people think this is important. The tipping point is when most oncologists in senior positions agree that we can and must speed up clinical research. Because we’re not there yet.”

## Limitations

It is critical to note three study limitations. It is likely that with a larger and more geographically diverse sample, additional barriers and facilitators would be identified, with greater fidelity and nuance. Furthermore, the sample of experts invited to participate was not randomized and was weighted more heavily towards participants from Canada and the United States, limiting the study's capacity to reflect on global opinions. Cultures of research and regulation can vary significantly nationally and regionally, which would further influence participants' interpretation of opportunities and barriers. To mitigate these limits, a three-round methodology for collecting expert opinion was implemented to increase robustness. At the same time, studies with more diverse and larger numbers of experts is needed, particularly given regional and national variations in access to new trial designs, real world data, and precision technologies, combined with the increased urgency for cross-site collaboration and more robust actions for planetary health.

## Data Availability

The raw data supporting the conclusions of this article will be made available by the authors, without undue reservation.
